# Transition Networks Unveil Disorder-to-Order Transformations in A*β* Caused by Glycosaminoglycans or Lipids

**DOI:** 10.3390/ijms241411238

**Published:** 2023-07-08

**Authors:** Moritz Schäffler, Suman Samantray, Birgit Strodel

**Affiliations:** 1 Institute of Biological Information Processing, Structural Biochemistry (IBI-7), Forschungszentrum Jülich, 52428 Jülich, Germany; mo.schaeffler@fz-juelich.de (M.S.); sumansamantray06@gmail.com (S.S.); 2 Institute of Theoretical and Computational Chemistry, Heinrich Heine University Düsseldorf, 40225 Düsseldorf, Germany

**Keywords:** intrinsically disordered proteins, molecular dynamics simulations, transition networks, amyloid-*β*, disorder-to-order transition

## Abstract

The aggregation of amyloid-β (Aβ) peptides, particularly of A$\beta_{1-42}$, has been linked to the pathogenesis of Alzheimer’s disease. In this study, we focus on the conformational change of A$\beta_{1-42}$ in the presence of glycosaminoglycans (GAGs) and 1-palmitoyl-2-oleoyl-sn-glycero-3-phosphocholine (POPC) lipids using molecular dynamics simulations. We analyze the conformational changes that occur in Aβ by extracting the key structural features that are then used to generate transition networks. Using the same three features per network highlights the transitions from intrinsically disordered states ubiquitous in A$\beta_{1-42}$ in solution to more compact states arising from stable β-hairpin formation when A$\beta_{1-42}$ is in the vicinity of a GAG molecule, and even more compact states characterized by a α-helix or β-sheet structures when A$\beta_{1-42}$ interacts with a POPC lipid cluster. We show that the molecular mechanisms underlying these transitions from disorder to order are different for the A$\beta_{1-42}$/GAG and A$\beta_{1-42}$/POPC systems. While in the latter the hydrophobicity provided by the lipid tails facilitates the folding of A$\beta_{1-42}$, in the case of GAG there are hardly any intermolecular A$\beta_{1-42}$–GAG interactions. Instead, GAG removes sodium ions from the peptide, allowing stronger electrostatic interactions within the peptide that stabilize a β-hairpin. Our results contribute to the growing knowledge of the role of GAGs and lipids in the conformational preferences of the Aβ peptide, which in turn influences its aggregation into toxic oligomers and amyloid fibrils.

## 1. Introduction

Intrinsically disordered proteins (IDPs) are a class of proteins that do not exhibit a well-defined three-dimensional structure in their native state. Instead, IDPs adopt an ensemble of different conformations, which allows them to perform a variety of functions, such as cell signaling, cell cycle control, and protein–protein interaction, but are also associated with a variety of disease pathways. IDPs are able to bind to a wide range of interaction partners and often undergo a disorder-to-order transition upon binding, which can lead to the formation of new structures and the initiation of interaction-specific functions [1,2]. One heavily studied example of an IDP is the amyloid-β peptide (Aβ), which is involved in the development of Alzheimer’s disease [3,4]. Aβ is a peptide that can form aggregates called amyloid plaques in the brain, which are thought to contribute to the cognitive decline associated with Alzheimer’s disease. As typical for an IDP, Aβ is able to bind to various interaction partners, resulting in conformational changes in Aβ. For example, in a previous study we showed that Aβ undergoes a disorder-to-order transition when in complex with three 1-palmitoyl-2-oleoyl-sn-glycero-3-phosphocholine (POPC) lipids [5].

Molecular examination of Aβ aggregate samples from Alzheimer’s disease affected patients have revealed a significant presence of charged polyelectrolytes, especially polysaccharides, belonging to the class of glycosaminoglycans (GAGs) [6]. GAGs are long chains of repeating disaccharide units and are found in various tissues, including cartilage and the extracellular matrix. They play a critical role in maintaining the structural integrity of these tissues and also act as lubricants and shock absorbers [7,8,9]. Their elevated presence in Aβ amyloid deposits suggests that GAGs may be involved in the formation and stability of Aβ plaques. Biophysical studies have shown that GAGs promote aggregation, nucleation, and formation of amyloid fibrils; however, the molecular details are not yet known [6].

Due to the wide conformational heterogeneity of IDPs such as Aβ, the experimental characterization of their structures is a challenging task [10,11]. Experimental techniques that can be applied to this goal usually average over a wide range of conformations, e.g., nuclear magnetic resonance (NMR) spectroscopy, small angle X-ray scattering (SAXS), cryo-electron microscopy, or single-molecule Förster resonance energy transfer (smFRET) spectroscopy. Consequently, the structural information obtained by these techniques is limited in the case of IDPs. Molecular dynamics (MD) simulations offer a complementary approach for gaining insights into the structural properties of IDPs, as they allow for the study of IDPs in a dynamic and spatiotemporal manner by simulating the motion of individual atoms over time, thus providing a molecular-level understanding of their conformational changes. Moreover, combining the MD approach with network-based models, such as Markov state models (MSMs) or conformational transition networks (TNs), yields a comprehensive understanding of the structural behavior of IDPs [12,13,14,15,16], as the networks reveal the underlying mechanisms of molecular processes that are hidden within the vast amounts of MD simulation data by generating human-interpretable networks that help to illustrate the molecular processes under investigation [17,18,19]. The TN approach pursued by our group is a solution for those who want a network model of protein motions captured by MD simulations, including explicit modeling of the protein’s environment, but do not need a method that sets up a master equation for the dynamics, since TNs rely purely on geometric clustering to extract the crucial features of protein conformational transitions (and not on kinetic clustering as in Markov state modeling) [20].

In this study we compare the conformational ensembles of Aβ under different external conditions: Aβ alone in solution [15], Aβ in contact with a small lipid cluster consisting of three POPC lipids [5], and Aβ in interaction with a GAG. For the GAG, we chose a polymer involving sixteen chondroitin-4-sulfate subunits, which are the alternating monosaccharides D-galactosamine (GalNAc) sulfated at position 4 and D-glucuronic acid (GlcUA), resulting in -GalNAc(4S)-β(1→4)-GlcUA-β(1→3)-. In all simulations, Aβ was modeled as the alloform having 42 amino acid residues (known as A$\beta_{1-42}$). To compare the conformational ensembles of Aβ under the different conditions, we created TNs based on 4 or 6 μs MD data per system. In order to obtain TNs that are comparable with each other, we used the same molecular features (or descriptors) to define the states of the underlying transition matrix. To capture the conformational changes and identify possible disorder-to-order transitions, we used the number of residues forming α-helical or β-sheet structures as well as the N-to-C distance of the peptide. The resulting TNs confirmed that Aβ in solution is an IDP that undergoes an unstructured-to-structured transition upon interaction with either the POPC cluster or the GAG. However, the causes of the emergence of structural order in Aβ are completely different, as shown here.

## 2. Results and Discussion

### 2.1. Transition Network of Aβ in Solution

**Figure 1:**
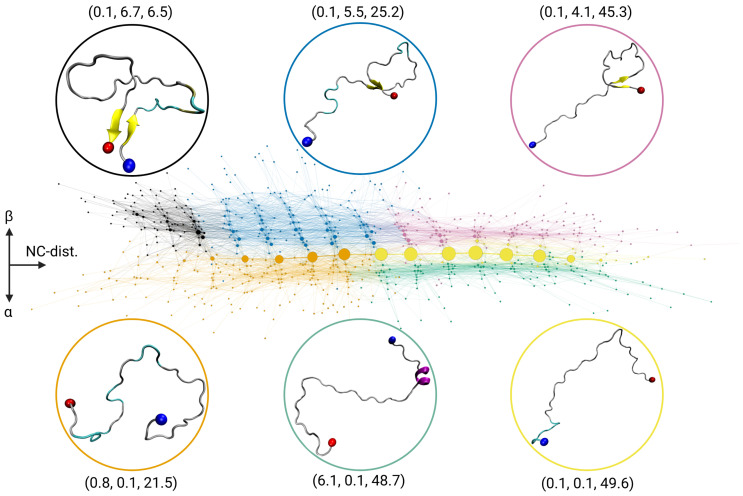
Transition network of the Aβ monomer in solution. For state assignment, three descriptors are used: (i) the number of residues forming α-helical structure ($N_{\alpha }$), (ii) the number of residues forming β-sheet structure ($N_{\beta }$), (iii) the N-to-C distance ($d_{\mathrm{NC}}$). The layout of the TN is such that $d_{\mathrm{NC}}$ increases from left to right along the *x*-axis, $N_{\beta }$ increases in positive *y*-direction, and $N_{\alpha }$ increases with negative *y*-direction. The nodes are colored according to their community membership, and the average descriptor values ($N_{\alpha }$, $N_{\beta }$, $d_{\mathrm{NC}}$) of the communities are given. For the highest-populated node per community, a representative structures is shown as cartoon (β-sheets in yellow, α-helices in purple) with the N- and C-termini being indicated by blue and red spheres, respectively.

The Aβ monomer in solution classifies as an IDP [21], thus its kinetics can be described by a flat free-energy surface that consists of many local minima that are separated by low-energy barriers [13,15]. In other words, the peptide does not exhibit a unique three-dimensional folded structure that would be connected to a surface-dominating minimum of low free energy, but has a low propensity towards forming α-helices or β-sheets. This behavior is very well reflected by the TN in Figure 1. The most populated states belong to the community of states colored in orange and yellow, which are aligned along the horizontal axis (called *x*-axis in the following). These two communities represent states that are primarily disordered with average descriptor values of (0.8, 0.1, 21.5) and (0.1, 0.1, 49.6), respectively, which means that there are neither α-helices nor β-sheets present in these Aβ conformations (first two descriptors) and the N-to-C distance varies, on average, between 21 and 50 Å. Thus, the most populated states only vary in their expansion, which increases from left to right in the TN, while they adopt random coil structures, reflecting the intrinsically disordered nature of the Aβ monomer in solution.

Communities shown in black with descriptor values (0.1, 6.7, 6.5), blue with (0.1, 5.5, 25.2), and pink with (0.1, 4.1, 45.3) represent the states with increasing amounts of β-sheets (second descriptor value) as they move toward the top of the network. In contrast, the states exhibiting α-helical structures are located towards the bottom of the network, and are contained within the community shown in green with descriptor values (6.1, 0.1, 48.7). However, the states containing β-sheets or α-helices are only sparsely populated compared to the completely random-coil communities shown in orange and yellow. This is even better seen from the distribution of the descriptor values, which are shown in Figure S1 along with the averages and variances in Table S1 in the Supplementary Materials. The population of the different communities is provided in Table S2.

In summary, the arrangement of the states of the TN allows to impose a coordinate system onto its layout, where the N-to-C distance is on the *x*-axis with increasing values from left to right, and the secondary structure is resolved along the *y*-axis, with disordered states at y≈0, increasing amounts of β-sheet structures for y>0, and increasing α-helical structures for y<0. It has to be stressed that this layout mainly arose from the *Force Atlas 2* optimization and was only slightly adjusted for visualization.

### 2.2. Transition Network of Aβ in the Presence of a GAG Molecule

**Figure 2:**
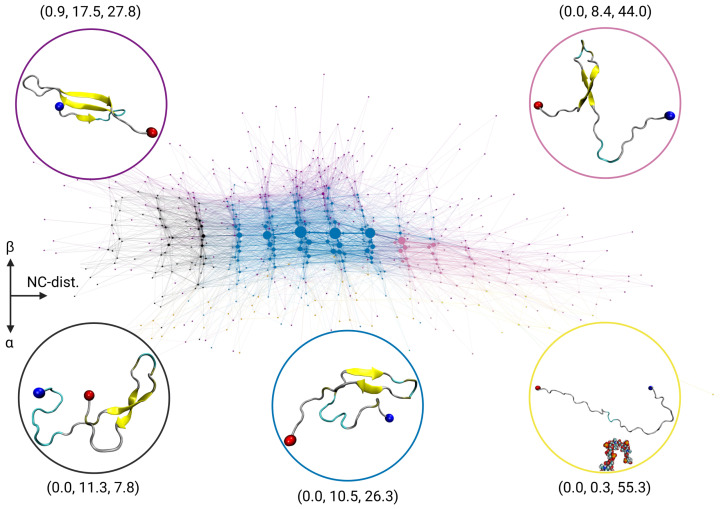
Transition network of the Aβ in the presence of a GAG molecule. The nodes are colored according to their community membership, and the average descriptor values ($N_{\alpha }$, $N_{\beta }$, $d_{\mathrm{NC}}$) of the communities and a representative snapshot are provided. The color of the communities was chosen as in Figure 1, so that states with the same or similar descriptor values are represented with the same color as in the Aβ-only system. See the caption of Figure 1 for further explanations of the graphical representation of the TN.

In order to study the conformational change of Aβ in the presence of a sulfated GAG compared to Aβ alone, we calculated the TN using the same three descriptors as before. The resulting TN is shown in Figure 2. Also the coloring of the communities has been chosen as for the Aβ monomer in solution to highlight the shift in community population. Thus, nodes that were displayed in a certain color in Figure 2 are displayed in that color again, with the descriptor values serving as the basis for the color assignment. However, depending on the existence and population of the various states, the relevant communities may appear in somewhat different parts of the TN than in the TN of the Aβ-only system. Nonetheless, the coordinate system is still the same, i.e., increasing N-to-C distances correspond to an increase along the horizontal axis, while states with increasing amounts of β-sheets are found in the positive *y*-direction, and in the opposite direction, there are very few sparsely populated states exhibiting α-helical structures.

The TN for the Aβ-GAG system looks quite different from that for the Aβ-only system. First, there is a drastic shift in the state population away from the disordered states, which are now presented by only one community, shown in yellow and with average descriptor values of (0.0, 0.3, 55.3). These disordered states are hardly populated with Aβ-GAG. Instead, the most populated states belong to the community shown in blue, which has average descriptor values of (0.0, 10.5, 26.3) and thus contains structures of intermediate compactness and a substantial amount of β-sheets. The community to the left shown in black with average descriptor values of (0.0, 11.3, 7.8) represents states with comparable β-sheet propensity, yet higher compactness. However, these states are only sparsely populated (Table S2). To the right of the blue-colored community, there is the community shown in pink with average descriptor values of (0.0, 8.4, 44.0), which also harbors β-sheet rich states, yet with large N-to-C distances. The population of this community is between those of the highest populated community shown in blue and the black-colored community. All three communities involve Aβ structures where about one fourth of the residues is part of a β-hairpin structure, as the representative structures in Figure 2 show, which mainly differ in the orientation of the N- and C-terminals with respect to each other. At the very top of the TN, mainly above the blue community, there are states combined into the community shown in purple, which are not present in the Aβ-only system. With average descriptor values of (0.9, 17.5, 27.6), these are states with even higher amounts of β-sheet structures. Here, about 42% of the Aβ residues are involved in β-sheet formation, which is accomplished by a β-sheet with three strands, where the third strand aligns with the previously mentioned β-hairpin.

**Figure 3:**
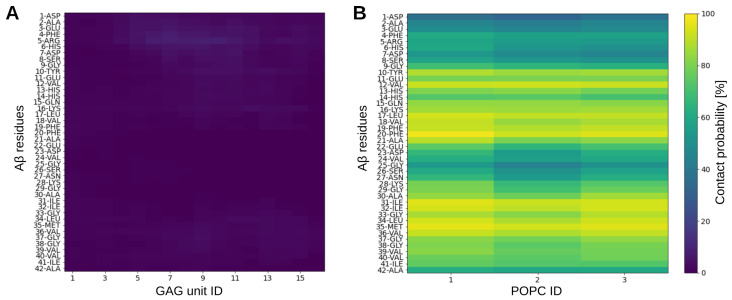
Intermolecular contact maps for Aβ interacting with (**A**) a GAG molecule and (**B**) three POPC lipids. The interactions are separated into residue–monosaccharide interactions for the Aβ-GAG system and residue–lipid interactions for the Aβ-POPC system. Two interaction partners were considered to be in contact if in a given frame of the trajectory they are closer than 10 Å. The resulting number of contacts were normalized with respect to the total number of time frames per trajectory, yielding a contact probability (see color scale on the right).

In summary, compared to the TN of the Aβ-only system, there is a significant population shift away from disordered states, towards states with considerable amounts of β-sheets (Figure S1 and Table S1), which results from the formation of a stable β-hairpin. This drastic change in secondary structure is astonishing, as Aβ was rarely in direct contact with the GAG, as the time-averaged distance matrix in Figure 3A shows. This molecular avoidance is understandable, given that both molecules are negatively charged (3− and 16−, respectively). Nevertheless, the contact map of Aβ-GAG interactions with populations shown only up to the maximum value of ≈8.5% (Figure S2) identify the positively charged Aβ residues Arg5 (in particular) and Lys16 as preferred binding sites for GAG. All other interactions that appear in the contact matrix simply result from their proximity to Arg5 and Lys16. The preference of GAG binding to arginine compared to lysine has been reported previously for other proteins [22]. However, these sparsely populated interactions are not sufficient to explain the GAG-induced structural changes in Aβ, and further reasons are given below.

### 2.3. Transition Network of Aβ Interacting with a POPC Cluster

**Figure 4:**
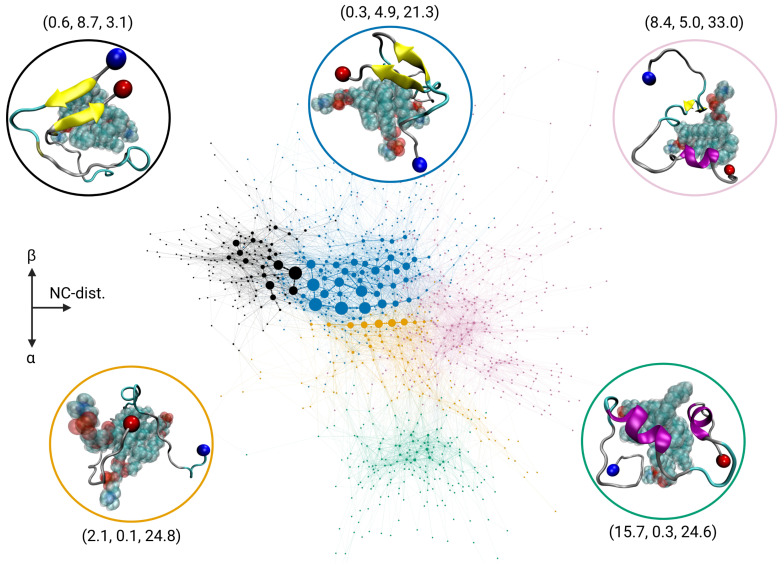
Transition network of Aβ in complex with three POPC lipids. The color of the communities was chosen as in Figure 1, so that states with the same or similar descriptor values are represented with the same color as in the Aβ-only system. However, the communities shown here in pink and green are not found in either the Aβ-only or Aβ-GAG systems. See the caption of Figure 1 for further explanations of the graphical representation of the TN.

To put the TN of the Aβ-GAG system into perspective, we also generated the TN of Aβ in interaction with three POPC lipids. In our previous study, we showed that under these conditions the Aβ monomer undergoes a disorder-to-order transition, which is facilitated by interactions between hydrophobic residues of Aβ and the lipid tails (Figure 3B) [5]. Here we use the simulations of that study to calculate the TN using the same three descriptors as before, resulting in the TN shown in Figure 4. The structures of the Aβ peptide in contact with POPC differ significantly from those of the Aβ-only system, resulting in many new states. For this reason, direct mapping of the color code of the communities was not possible. Nevertheless, the coloration was chosen to be as close as possible to the previous representations. Again, the communities were distributed along the three axes $d_{\mathrm{NC}}$ (positive *x*-axis), $N_{\beta }$ (positive *y*-axis), and $N_{\alpha }$ (negative *y*-axis).

As for the Aβ-only system, there is a community colored orange that harbors disordered states with average descriptor values of (2.1, 0.1, 24.8). The underlying states have slight deviations in their N-to-C distance, with increasing values from left to right. However, they are less expanded than the states of the yellow-colored community found for the Aβ-only and Aβ-GAG systems, which is not present here. Moreover, some of the Aβ-POPC states belonging to the orange-colored community feature some α-helical structure, which is also different from the corresponding Aβ-only community. These states are located towards the bottom of the community, since, as before, the *y*-axis distinguishes between α-helical and β-sheet structures. Another difference is that the disordered states are only sparsely populated in the Aβ-POPC system. Here, the two most populated communities are the ones colored black and blue, which have average descriptor values of (0.6, 8.7, 3.1) and (0.3, 4.9, 21.3), respectively, and thus mainly contain compact states with considerable β-sheet content. On the opposite side along the *y*-axis, at the bottom of the TN, there is a distinct cluster of states shown in green with average descriptor values of (15.7, 0.3, 24.6). This community exhibits high amounts of α-helical structures. These states are infrequently visited, but their spatial separation, determined using the *Force Atlas 2* algorithm based on their low connectivity with other communities, suggests that this community corresponds to a local minimum in the free-energy landscape, separated from the other communities by a high-energy barrier. The rightmost community along the *x*-axis, colored pink, exhibits the states with the largest N-to-C distance of the peptide in the Aβ-POPC system, as indicated by the average descriptor values of (8.4, 5.0, 33.0). The first two descriptors indicate that the states of this community feature both an α-helix and a β-sheet. This structural finding, as also the location of this community in the TN, suggests that these states are intermediates between the β-sheet-rich states of the blue community and the α-helical-rich states of the green community. The fact that the pink community is also next to the orange community results from the projection of the TN onto a two-dimensional plane. In a third dimension, the orange community would appear closer to the viewer.

Comparison of this TN to the TN of the Aβ-only system highlights the conformational change of Aβ towards folded states when in complex with lipids, as also revealed by the descriptor distributions (Figure S1 and Table S1) and community populations (Table S2). One can see a notable shift in the state population towards more compact states; the current TN has its maximum extension in the *y*-direction, while for the Aβ-only system the maximum TN extension is in the *x*-direction. Such a change in TN geometry did not occur in the Aβ-GAG system where Aβ remained very expanded and only adopted β-sheet structures but not α-helices. The change of TN geometry for the Aβ-POPC system also reveals a change in the underlying free-energy surface, from being rather flat with many local minima (Aβ-only and Aβ-GAG) towards multiple definite energy basins of (semi-)folded Aβ conformations.

### 2.4. Interactions in the Aβ-GAG System

In order to understand the conformation switching of Aβ in presence of the GAG, we investigated their molecular interactions as well as the impact of the GAG on the water dynamics and Na${}^{+}$ distribution. As already mentioned, the GAG induces the structural change in Aβ with hardly any direct interactions between the peptide and the GAG. Thus, the mode of interaction differs notably to that of the Aβ–POPC interactions, as the comparison between the two contact maps in Figure 3 shows. In the case of the Aβ-GAG system, only some of the positively charged side chains of Aβ, in particular Arg5 and the neighbored residue His6, are in direct contact with the GAG for about 9% of the time. In contrast, the contact map of the Aβ-POPC system shows that some of the hydrophobic residues interact with POPC during the whole course of the simulation, which causes the conformation switching in Aβ [5].

Since Aβ and the GAG do not interact directly with each other, the effect of the GAG on the peptide must therefore be indirect. We considered two possibilities for this, which both could arise due to the high negative charge of the GAG: (i) a change in the water ordering and dynamics, and (ii) a change in the Na${}^{+}$ ion distribution. To address (i), we determined the water structure in close proximity to Aβ using the translational and orientational order parameters *T* and *Q* (see Equations (2) and (3)). Table 1 shows the ensemble and time average for both quantities considering all water molecules in the vicinity (i.e., within 10 Å) of the Aβ peptide. As one can see, the order parameters for the water surrounding Aβ show no significant difference between the Aβ-only and Aβ-GAG simulations. This also applies to the water around the GAG molecule. Furthermore, the results did also not change considerably when reducing the radius of the water molecules to be considered in the calculation to 5 Åwithin the solutes. To probe the dynamics of the water molecules, we used the lifetimes of the H-bonds formed between water and either Aβ or the GAG (see Equations (4) and (5)). The results, also listed in Table 1, show that in either system and for either molecule, the values for the stretching exponent β are about 0.5, reflecting that the relaxation of the H-bonds deviates from exponential behavior, which is caused by interactions between water and Aβ or the GAG. The deviation is strongest for the GAG and smallest for Aβ in the Aβ-GAG system, which suggests that the presence of the GAG molecule weakens the Aβ-water interactions. However, the differences in the water dynamics between the molecules are only minor, as confirmed by the lifetimes of the H-bonds. Interestingly, the lifetime of the H-bonds formed between the GAG and water is an intermediate of that of Aβ in different environments, i.e., the high negative charge of the GAG does not slow down the water dynamics. Overall, we did not find noteworthy effects of the GAG on the water structure and dynamics around Aβ that would explain its drastic change in conformation.

**Figure 5:**
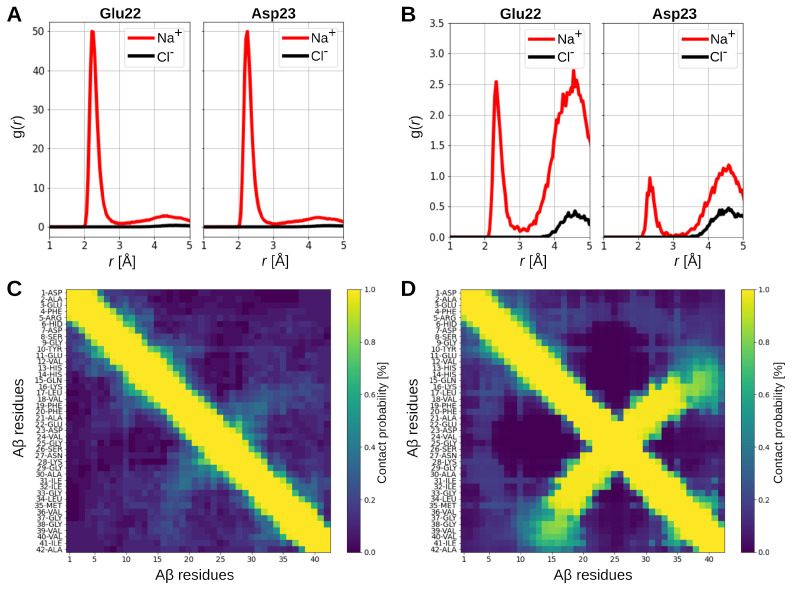
The radial distributions g(r) of Na${}^{+}$ (red) and Cl${}^{-}$ (black) relative to the carboxyl groups of the side chains of Glu22 and Asp23 of Aβ in (**A**) the Aβ-only system and (**B**) the Aβ-GAG system. The intrapeptide contacts between the Aβ residues in (**C**) the Aβ-only system and (**D**) the Aβ-GAG system. Two interaction partners were considered to be in contact if in a given frame of the trajectory they are closer than 10 Å. The resulting number of contacts were normalized with respect to the total number of time frames per trajectory, yielding a contact probability.

To assess whether the negative charge has considerable effects on the distribution of the ions in the system, we calculated the radial distributions, g(r), of Na${}^{+}$ and Cl${}^{-}$ with respect to the negatively and positively charged Aβ residues in the Aβ-only and Aβ-GAG system. For the latter, we also determined the ion distribution around the GAG molecule. While the Cl${}^{-}$ distributions are not noteworthily affected by the GAG, the Na${}^{+}$ distribution changed dramatically. Panels A and B of Figure 5 show the results for the carboxyl groups of residues Glu22 and Asp23 for Aβ in the two systems, while all other distributions are shown in Figures S3–S6 in the Supplementary Materials. Comparison of these distributions reveals that in the Aβ-GAG system, the interaction between these two residues and the surrounding sodium ions is an order of magnitude smaller compared to the simulation of Aβ alone. This is a result of the strong attraction between the negatively charged COO${}^{-}$ and OSO${}_{3}^{-}$ groups of the GAG and Na${}^{+}$ (Figure S7, discussed in detail in our previous work on GAGs [23]), making the peptide a less favorable interaction partner. The withdrawal of Na${}^{+}$ from Aβ descreens the electrostatic interactions between the peptide residues, which enables a strong attraction between Glu22/Asp23 and Lys28. This is visible from intrapeptide residue–residue contact maps shown in Figure 5C,D for the A*β*-only and Aβ-GAG system, respectively. The salt bridge between Glu22/Asp23 and Lys28 in the Aβ-GAG system results in a β-hairpin, which was already mentioned when discussing the TN of that system, which shows as strong contacts perpendicular to the diagonal that reflect the neighboring contacts along the sequence. In the Aβ-only system, such a perpendicular trace of contacts is only slightly visible, resulting from short-lived interactions. Otherwise the peptide is devoid of interactions beyond i,i+3 along the sequence (*i* referring to the residue number), which is in line with the observation from the TN of the Aβ-only system.

In summary, the effect of the GAG on Aβ results from notably changing the distribution of the sodium ions in the vicinity of the peptide, as these ions are strongly attracted by the GAG. As a consequence, the formation of intrapeptide salt bridges is facilitated, in particular between residues Glu22/Asp23 and Lys28, which is further stabilized by subsequent β-hairpin formation. There are no notable direct interactions between the GAG and Aβ nor relevant effects of the GAG on the water order and dynamics, which could serve as alternative explanations for the conformational changes in Aβ in the presence of the GAG.

### 2.5. Discussion

First, we discuss the TN of the Aβ-only system, which is a prime example of what one would expect the TN of an IDP to look like. This TN is characterized by the presence of many states, most of which have small populations and many connections to other states, corresponding to a broad but flat free-energy landscape. Furthermore, the vast majority of states belongs to Aβ conformations that are purely random coils and vary only in their spatial expansion. Upon running the *Force Atlas 2* optimization algorithm of Gephi, the states differing mainly in their N-to-C distance aligned along one axis, which we assigned as the *x*-axis. The second axis in our TN representation, the *y*-axis, turned out to represent the change in secondary structure, with positive *y*-values being associated with more β-sheet structure and negative *y*-values with more α-helical structures. Translating the TN to a free-energy surface, one can conclude that the peptide populates a shallow energy basin corresponding to random-coil structures and large N-to-C distance variations. Free-energy states with a defined secondary structure are rarely visited and quickly return to the disordered states.

The TN of Aβ together with a GAG differs significantly from that of Aβ alone. The TN was calculated using the same three descriptors as before, and the automatically assigned layout of the nodes along the *x*- and *y*-axes did not change either. The TN clearly revealed that Aβ undergoes a structural change in the presence of the GAG. While Aβ can still adopt fully extended structures in the presence of the GAG, the most populated states are found for more compact and more β-sheet rich structures. These structures contain a very stable β-hairpin maintained by the presence of a salt bridge between Glu22/Arg23 and Lys28, which is made possible by a descreening of the electrostatic interaction between these residues due to a shift in the local sodium ion concentration away from the peptide. These ions are instead attracted to the strongly negatively charged GAG. This distinct structural switching of Aβ in the presence of a GAG agrees with the experimental finding that in the presence of GAGs, the random-coil to amyloidogenic β-sheet transition of Aβ is accelerated, leading to a more rapid fibril formation [6]. This observation is of biological relevance because GAGs are important components of the extracellular space, where they can be located on the cell surface or within the extracellular matrix. There, they exist in two forms: covalently attached to the protein core of proteoglycans or as independent macromolecules. Studies have revealed a close connection between GAGs and amyloid fibrils extracted from humans. Evidence indicates that GAGs actively participate in the formation and stabilization of amyloid fibrils [9,24,25]. Here, we show for the first time how GAGs can cause a structural change in Aβ from a random coil to a β-sheet structure. This structural change is even more remarkable considering that Aβ and the GAG show almost no direct interaction with each other and only GAG-induced changes in the local peptide environment trigger a conformational change in Aβ.

Some of these observations should be investigated in further studies. We chose the protonation state of Aβ to correspond to the physiological conditions of the extracellular space, i.e., about pH 7.4. At this pH, the three histidine residues of Aβ can be assumed to be neutral (the pK${}_{a}$ value of the free His is 6.0). On the other hand, the local environment of His6, His13, and His14 can change their pK${}_{a}$ value. Moreover, it is known that aging and Alzheimer’s disease can decrease the pH of the extracellular brain space to below 7 [26]. Therefore, it would be justified to simulate other protonation states of Aβ as we have already carried out for Aβ in solution [27,28], which resulted in random coil to β-sheet formation at the isoelectric point of Aβ (pI of 5.3) due to altered intrapeptide electrostatics. We expect that modeling the histidine residues as His${}^{+}$, which would yield neutral Aβ, would increase the interaction between the GAG and Aβ. This in turn would increase the local Na${}^{+}$ concentration around Aβ since it would now be in close proximity to the GAG molecule, while neutral Aβ would have a higher preference to form β-sheets. It will therefore be interesting to observe how these different forces affect the behavior of the peptide. Moreover, the observation that the GAG-induced decrease in sodium ion concentration near Aβ leads to structural changes in the peptide should also be further investigated by a titration simulation in which the concentration (and also the type of salt) is gradually increased, starting at zero, to determine the dependence of the Aβ structure on the salt. It is known from experiments that both the type and concentration of salts, particularly the type of cation, have significant effects on the rate of aggregation and the morphology of the resulting Aβ fibrils [29,30], but the effects of the salts on the peptide monomer are still unknown.

The TN of Aβ interacting with three POPC lipids also revealed a disordered-to-ordered transition in Aβ. However, certain differences exist compared to the Aβ-GAG system. First, Aβ forms a complex with the lipids over the whole time of the simulation. This leads to more compact peptide structures; fully extended structures did not occur anymore. Second, Aβ can also adopt α-helical structures and not only β-sheet-rich states. Either folded state is facilitated by hydrophobic interactions between the peptide and the lipids. The TN revealed that the α-helical structures populate a community that is somewhat separated from the other communities, which allowed us to conclude that the α-helical states correspond to a separate local minimum in a multi-funnel free-energy landscape. Thus the interaction with the POPC lipids not only shifts the main energy basin towards more compact configurations with structure formation, but also leads to a rougher free-energy landscape. The results of these simulations are consistent with a large body of experimental studies that attest to a central role for lipids in amyloid aggregation and disease development. Lipids are an integral component of many amyloid deposits in vivo, where their presence can influence fibril nucleation, morphology, and mechanical properties [31]. It was demonstrated that the toxicity of Aβ aggregates correlates with the amount of their β-sheet content, which, in turn, is increased by lipids present during Aβ aggregation [32]. With respect to helical Aβ structures, this appears to be the prerequisite for membrane incorporation of Aβ [33,34,35,36,37].

## 3. Materials and Methods

### 3.1. Molecular Dynamics Simulations

In all simulations, Aβ was simulated as A$\beta_{1-42}$ with the histidine residues modeled as neutral and no terminal capping groups, leading to an overall peptide charge of 3−. The three systems of only Aβ, Aβ plus GAG, and Aβ with three POPC lipids (henceforth called Aβ-only, Aβ-GAG, and Aβ-POPC, respectively) were simulated using the GROMACS simulation package [38]. Since two of the three systems were originally part of different studies [5,15,19], some of the MD simulation settings (but not the force field parameters and ion concentration) differ slightly between the simulations. While this is not ideal, these differences appear to be negligible given the remarkable results presented below. In each simulation, the Aβ peptide was modeled using the CHARMM36m force field [39]. It has been found in previous studies that the CHARMM36m force field is best suited for modeling both monomeric Aβ[15] as well as amyloid aggregation [40]. CHARMM36m is a polypeptide force field that can be combined with the original force field CHARMM36 [41] for modeling the POPC lipids. For the GAGs, we used the parameters as available through the Glycan Reader & Modeler module [42,43,44] of the CHARMM-GUI web server [45], as in our previous studies of GAGs alone and in interaction with multiple A$\beta_{16-22}$ peptides [23,46]. The preparation of the systems followed the same standard protocol: First the solute(s) were placed in the simulation box, which was then filled with TIP3P water molecules [47] together with Na${}^{+}$ and Cl${}^{-}$ ions to neutralize the systems and achieve a physiological salt concentration of 150 mM. After equilibration of the systems, each of them was simulated under NpT conditions at 1 bar, which was accomplished using a Parrinello–Rahman pressure coupling scheme [48]. The simulations of Aβ-GAG and Aβ-POPC were carried out at 310 K using a Nosé–Hoover thermostat [49,50], while the Aβ system was simulated at 300 K using a velocity rescaling thermostat [51]. In the case of the Aβ-GAG and Aβ-POPC, the simulations were carried out for 4 μs involving 1 × 4 μs and 2 × 2 μs, respectively, while the Aβ system was simulated for 1 × 6 μs. All simulations were achieved under periodic boundary conditions in all directions and the particle-mesh Ewald method [52] was used for calculating the electrostatic interactions. The cutoffs for van der Waals and Coulomb interaction calculations in real space were both set to 12 Å. The minimum distance between any solute atom and any face or edge of the simulation box was set to 12 Å. All MD simulations were run on the supercomputer JURECA [53].

### 3.2. Transition Networks

In order to construct a TN, one chooses a set of *n* features that describe the process under study. These features are evaluated by descriptor functions $\left\{f_{i}\right\}$ that act on a given conformation x(t) and project the 3N-dimensional phase space onto an *n*-dimensional state S(t)

(1) $$x\left(t\right)\mapsto S\left(t\right)=[f_{1}\left(x\left(t\right)\right),f_{2}\left(x\left(t\right)\right),\;\ldots \;,f_{n}\left(x\left(t\right)\right)].$$

Here, x(t) is the conformation of an MD simulation at time *t* and *N* is the number of particles within the conformation. A crucial point in constructing a TN is the choice of descriptor functions. As discussed in our previous work [19,20], the number and type of descriptors have a huge impact on the resulting TN. While the choice of the type of descriptor functions is closely related to the process under study, the ideal number of descriptors is often up to trial and error. Choosing fewer descriptors yields a simpler TN, though more information is lost due to the projection onto a low-dimensional space. On the contrary, choosing more descriptors might yield a TN that is too complex to be intuitively interpreted. Here we decided to use the same three descriptor functions for the studied systems, which allows easy comparison between them. To describe the process of conformation switching of Aβ, we chose (i) the number of residues adopting an α-helical structure ($N_{\alpha }$), (ii) the number of residues adopting a β-sheet structure ($N_{\beta }$), and (iii) the distance between N- and C-terminus, called the N-to-C distance ($d_{\mathrm{NC}}$, in Å) as a measure of compactness of the peptide. The TNs were calculated with ATRANET, which is a Python package developed by our group. The software has been optimized to handle large amounts of MD data with many different descriptor functions to choose from, while still providing a dynamic framework to easily add custom descriptor functions [19,20]. ATRANET is available at https://github.com/strodel-group/ATRANET (accessed on 1 May 2023).

The transition matrix created by ATRANET can be visualized with the network analysis and visualization software Gephi 0.10 [54,55]. For the layout of the networks we chose the *Force Atlas 2* algorithm, which is a force-driven algorithm that takes into account the connectivity of pairs of nodes and their relative degree. As a result, nodes that have more transitions between them are displayed closer to each other. Thus, a large spacial separation in the layout of the TN corresponds to a large distance between the respective states in the high-dimensional phase space. Additionally, we chose the size of the nodes to be proportional to their diagonal entries in the transition matrix as a state with more self-transitions is more stable (i.e., has a lower free energy), while nodes with fewer self-transitions reflect higher-energy states. For visualization purposes, the depicted sizes are adjusted based on the minimum and maximum values for each network on a linear scale from 1 to 10. In the following, the size of the nodes will also be referred to as the population of the states. In addition, we used Gephi’s modularity class feature to divide the network into local communities, which makes it easier to identify groups of nodes that are strongly connected and have high similarity between states. In terms of a free-energy perspective, the states of a community belong to the same energy basin of an underlying multi-funnel free-energy landscape [56].

### 3.3. Analysis of Water around the Solutes

To elucidate possible effects of the water solvating the peptide or the GAG, we analyzed the water structure and dynamics around the solutes. For determining the water structure, we used the translational order parameter *T* and orientational order parameter *Q* [57]. The translational order parameter is given by

(2) $$T=\frac{1}{\zeta_{c}}\int_{0}^{\zeta_{c}}\left|g\left(\zeta \right)-1\right|d\zeta$$

where *g* refers to the oxygen–oxygen radial distribution function (RDF) and $\zeta =r\cdot \rho^{1/3}$ is a dimensional variable dependent on the radial distance *r* and the density of the water-oxygen atoms ($\rho =N_{O}/V$). The parameter $\zeta_{c}=2.8$ is chosen such that $g(\zeta_{c})∼0$. The order parameter *T* can be used to measure whether or not long-range interactions are present in a medium. For an ideal gas, the RDF is equal to 1 and hence T=0. In the case of a crystal, the RDF is different from 1 even for large distances, so *T* is large in a system with long-range order. The orientational parameter *Q* measures the ability of neighboring water molecules to produce tetrahedral arrangements. It is given by

(3) $$Q=1-\frac{3}{8}\sum_{j=1}^{3}\sum_{k=j+1}^{4}{\left(\cos\psi_{jk}+\frac{1}{3}\right)}^{2}$$

where $\psi_{jk}$ is the angle between neighboring O atoms *j* and *k* with central atom *i*. The value of *Q* can range from 0 to 1, where 0 corresponds to a random distribution of water molecules and 1 to a perfect tetrahedral arrangement.

To probe the dynamics of the water in the first solvation shell, we made use of the lifetimes of the hydrogen bonds (H-bonds) between water and the solutes (Aβ or GAG). To this end, we performed short simulations of 100 ns for the Aβ and Aβ-GAG system, using the same parameters as described above but writing out data every 0.5 ps to resolve the H-bond lifetimes. These simulations were analyzed in terms of the H-bond existing function $h(t_{0}+t)$, which is either 1 or 0 at a given time, depending on whether or not a specific H-bond is present. To improve statistics, multiple time origins $t_{0}$ are used in the calculation and the average is taken over all time origins and possible H-bonds. The mean H-bond lifetime 〈τ〉 can then be determined by calculating the autocorrelation function of the averaged $h(t_{0}+t)$ and fitting a stretched exponential function to it:

(4) $$c\left(t\right)=\exp\left\{{\left(-\frac{t}{\tau }\right)}^{\beta }\right\}$$

where c(t) refers to the autocorrelation function, τ is the lifetime, and β is the stretching factor. From this, the mean lifetime 〈τ〉 can be calculated via integration, which is analytically solved by the gamma function Γ:

(5) $$\langle \tau \rangle =\frac{\tau }{\beta }\Gamma \left(\frac{1}{\beta }\right)$$

## 4. Conclusions

We constructed the transition networks (TNs) revealing the conformational preferences and conversions of the amyloid β-peptide A$\beta_{1-42}$ (here simply called Aβ) under different conditions: as a single peptide in solution, Aβ in the presence of the GAG chondroitin-4-sulfate with sixteen subunits, and Aβ in complex with three POPC lipids. For defining the states of each TN, we chose the same three descriptors: (i) the number of residues with an α-helical structure, (ii) the number of residues with a β-sheet structure, (iii) the peptide distance from end-to-end. Using the same descriptors allows direct comparison of the resulting TNs and identification of changes in the underlying free-energy surfaces between the different systems. Moreover, the choice of a low-dimensional projection of the phase space due to using only three descriptors allows a very intuitive interpretation of the resulting TNs and directly visualizes the conformation switching of Aβ. In particular, we have shown how the interaction of Aβ with a GAG or POPC lipids leads to a transition from disorder to order of the intrinsically disordered monomer. Taking advantage of the similarities of the transition network layout, we can infer a shift of the main basin of the underlying free-energy surface from disordered conformations with large end-to-end separations to more compact conformations with high amounts of β-sheet. The overall increase in β-sheet-rich structures could, in turn, serve as a nucleus for amyloid aggregation and the formation of toxic oligomers. Our findings contribute to the growing body of knowledge on the role of GAGs and lipids in Aβ aggregation and the development of Alzheimer’s disease.

## Figures and Tables

**Table 1:** Translational and rotational order parameters *T* and *Q* of water in the vicinity (<10 Å) of Aβ and the GAG molecule, as well as the average stretching exponents β and mean lifetimes 〈τ〉 of H-bonds formed between water and Aβ or the GAG.

Molecule	*T*	*Q*	β	〈τ〉/[ps]
Aβ-only	0.37	0.44	0.555	16.45
Aβ in Aβ-GAG	0.34	0.44	0.565	14.54
GAG in Aβ-GAG	0.34	0.38	0.499	15.39

## Data Availability

The code for the analysis of the MD simulations is available at https://github.com/strodel-group/ATRANET (accessed on 1 May 2023). The trajectory files are available from the authors upon request.

## Supplementary Materials

The supporting information can be downloaded at: https://www.mdpi.com/article/10.3390/ijms241411238/s1.
