# Supplementary material for: Transition Networks Unveil Disorder-to-Order Transformations in Aβ Caused by Glycosaminoglycans or Lipids
Source: Int J Mol Sci. 2023 Jul 8;24(14):11238. doi: 10.3390/ijms241411238 (PMC10380057; doi:10.3390/ijms241411238)
Supplement: Supplementary file 1 [file ijms-24-11238-s001.zip › ijms-2439486-supplementary.pdf]

Supplementary Materials: Transition Networks Unveil Disorder-to-Order Transformations in Aβ Caused by Glycosaminoglycans or Lipids

Moritz Schäffler[1,2], Suman Samantray[1] and Birgit Strodel [1,2,*]

[1] Institute of Biological Information Processing: Structural Biochemistry (IBI-7), Forschungszentrum Jülich, 52428 Jülich, Germany
[2] Institute of Theoretical and Computational Chemistry, Heinrich Heine University Düsseldorf, 40225 Düsseldorf, Germany
* Correspondence: b.strodel@fz-juelich.de

Supplementary Tables

	N_α	N_β	d_{NC}/[Å]
Aβ only	0.8±1.9	2±3	34±17
Aβ in Aβ-GAG	0.2±0.7	10±4	28±11
Aβ in Aβ-POPC	3±5	5±4	20±12

Table S1. Mean and variance of the three descriptors used for the transitions networks of the Aβ-only, Aβ-GAG, and Aβ-POPC systems. The descriptors are: i) the number of residues forming α-helical structure (N_α), ii) the number of residues forming β-sheet structure (N_β), iii) the N-to-C distance (d_{NC}).

Community	Orange	Yellow	Black	Blue	Pink	Other
Aβ-only	20.0%	40.2%	8.7%	19.7%	7.1%	4.3%
Aβ-GAG	0.1%	1.0%	8.3%	70.3%	16.7%	3.6%
Aβ-POPC	13.1%	-	22.3%	54.9%	4.5%	5.2%

Table S2. Population of the transition network communities (identified by color) of the three Aβ systems.

Supplementary Figures

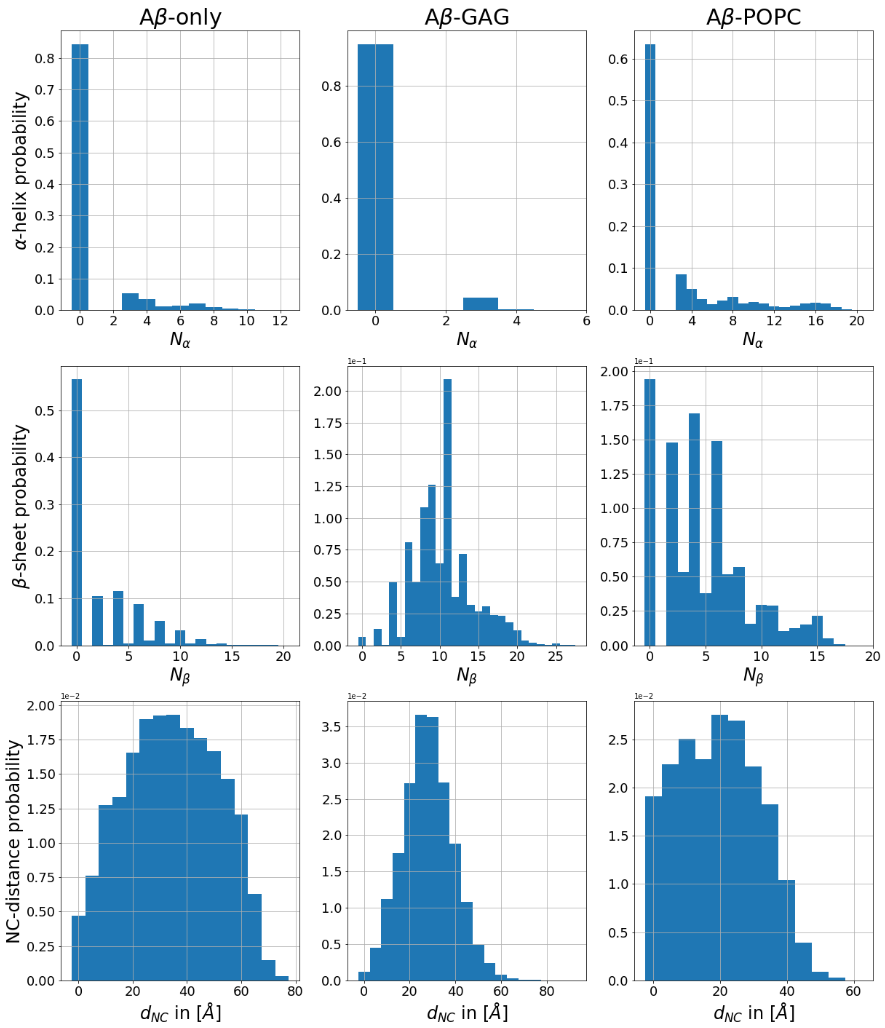

Figure S1. Probability distributions for the three descriptors used in the transitions network of the Aβ-only system (left), Aβ-GAG system (center), and Aβ-POPC system (right). The descriptors are: i) the number of residues forming α-helical structure (N_α, top), ii) the number of residues forming β-sheet structure (N_β, middle), iii) the N-to-C distance (d_{NC}, bottom).

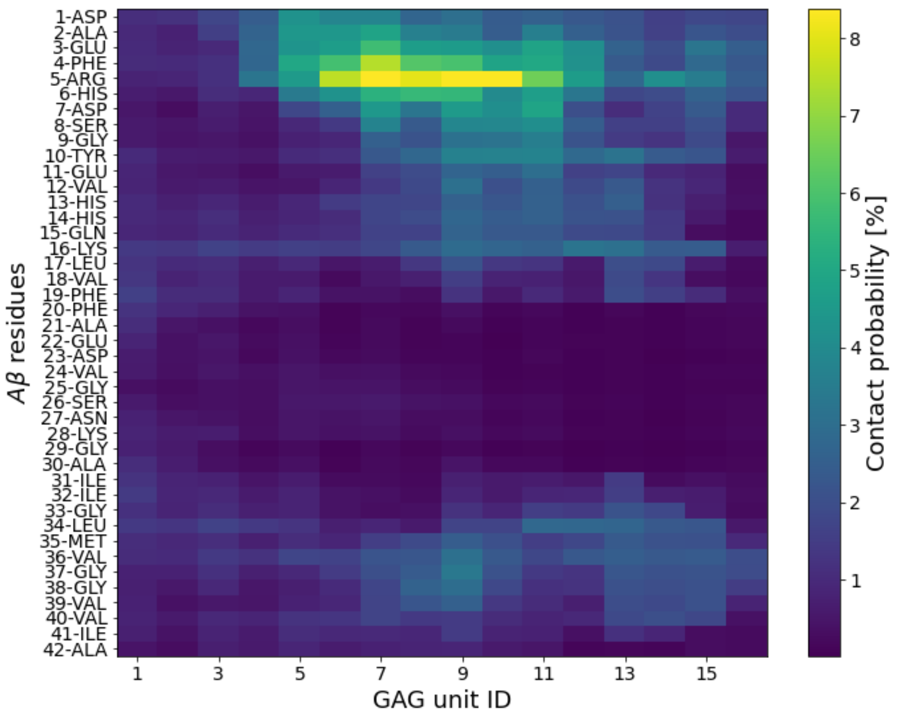

Figure S2. Intermolecular contact maps for Aβ interacting with a GAG molecule. The interactions are separated into residue–monosaccharide interactions. Two interaction partners were considered to be in contact if in a given frame of the trajectory they are closer than 10 Å. The resulting number of contacts were normalized with respect to the total number of time frames per trajectory, yielding a contact probability with a maximum value of about 8.5% (see color scale on the right).

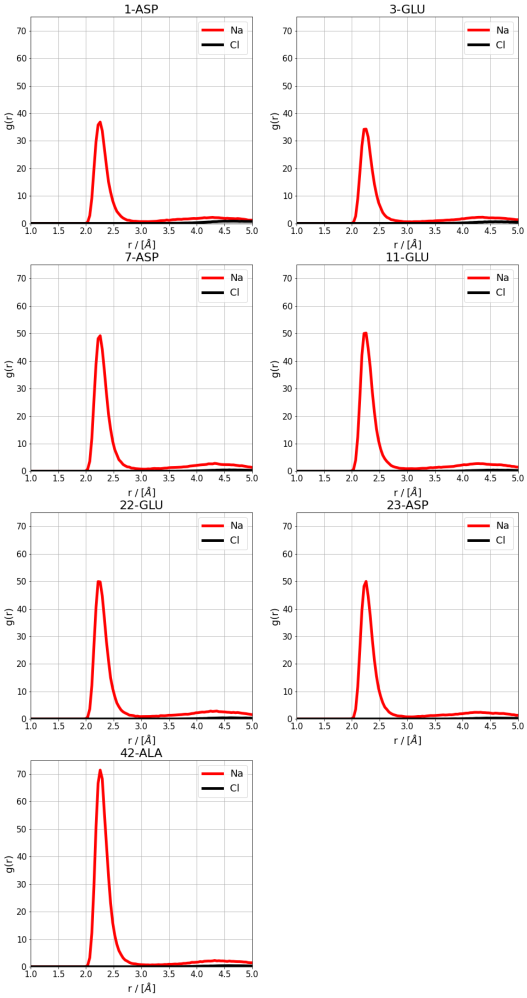

Figure S3. The radial distribution $g(r)$ of Na^+ (red) and Cl^- (black) relative to the carboxyl groups of negatively charged Aβ residues in the Aβ-only system.

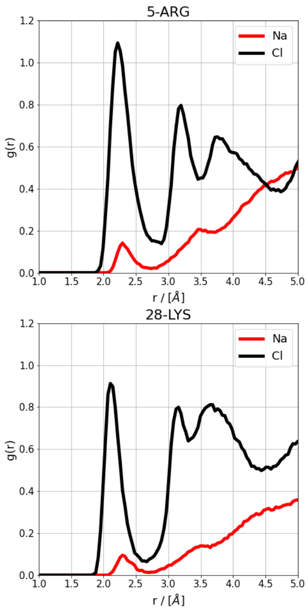

Figure S4. The radial distribution $g(r)$ of Na^+ (red) and Cl^- (black) relative to the charged groups of positively charged Aβ residues in the Aβ-only system.

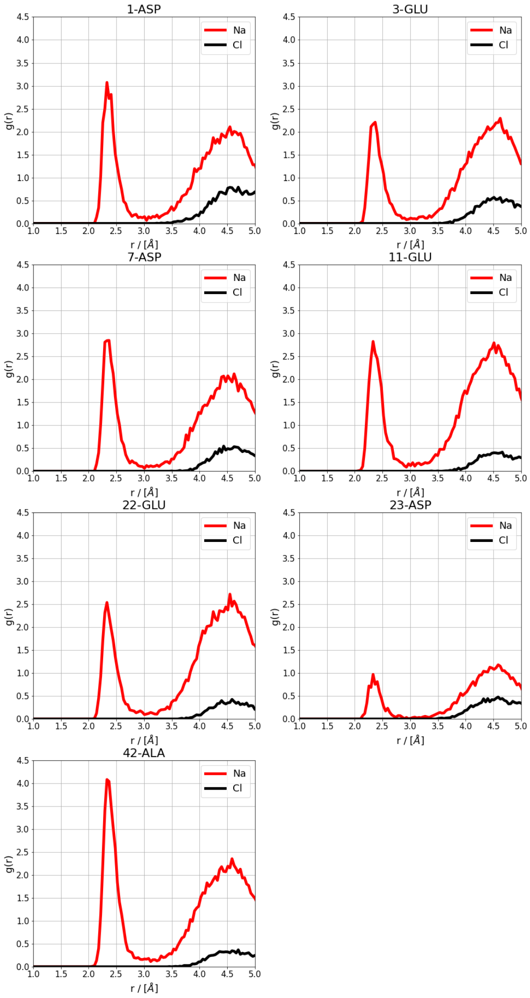

Figure S5. The radial distribution $g(r)$ of Na^{+} (red) and Cl^{-} (black) relative to the carboxyl groups of negatively charged Aβ residues in the Aβ-GAG system.

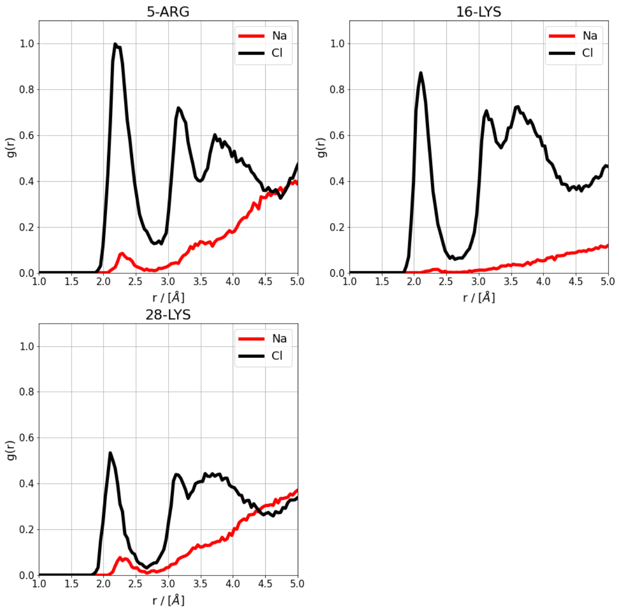

Figure S6. The radial distribution $g(r)$ of Na^+ (red) and Cl^- (black) relative to the charged groups of positively charged Aβ residues in the Aβ-GAG system.

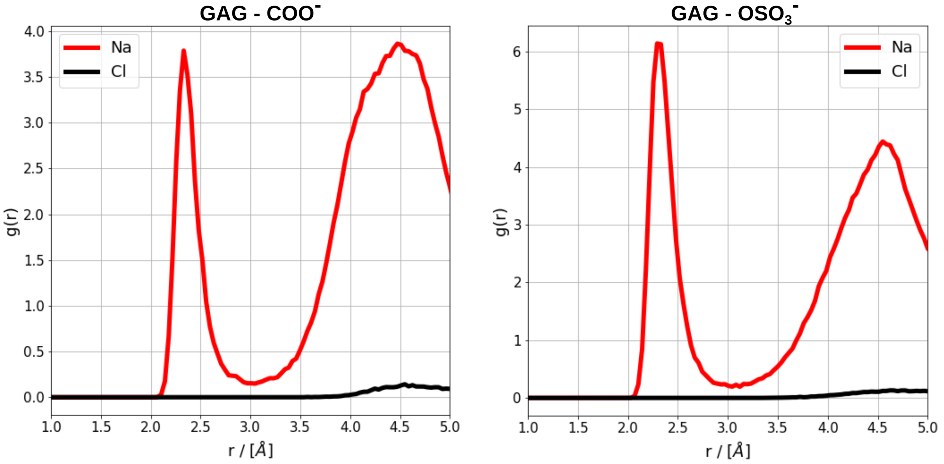

Figure S7. The radial distribution $g(r)$ of Na^+ (red) and Cl^- (black) relative to the COO^- (left) and OSO_3^- (right) groups of the GAG in the Aβ-GAG system.
